# Signaling Pathways in Melanogenesis

**DOI:** 10.3390/ijms17071144

**Published:** 2016-07-15

**Authors:** Stacey A. N. D’Mello, Graeme J. Finlay, Bruce C. Baguley, Marjan E. Askarian-Amiri

**Affiliations:** 1 Department of Molecular Medicine and Pathology, University of Auckland, Faculty of Medical and Health Sciences, University of Auckland, 85 Park Rd. Grafton, Auckland 1023, New Zealand; s.dmello@auckland.ac.nz (S.A.N.D.); g.finlay@auckland.ac.nz (G.J.F.); 2 Auckland Cancer Society Research Centre, University of Auckland, Faculty of Medical and Health Sciences, University of Auckland, 85 Park Rd. Grafton, Auckland 1023, New Zealand; b.baguley@auckland.ac.nz

**Keywords:** melanogenesis, signaling pathways in melanogenesis, MITF, tyrosinase

## Abstract

Melanocytes are melanin-producing cells found in skin, hair follicles, eyes, inner ear, bones, heart and brain of humans. They arise from pluripotent neural crest cells and differentiate in response to a complex network of interacting regulatory pathways. Melanins are pigment molecules that are endogenously synthesized by melanocytes. The light absorption of melanin in skin and hair leads to photoreceptor shielding, thermoregulation, photoprotection, camouflage and display coloring. Melanins are also powerful cation chelators and may act as free radical sinks. Melanin formation is a product of complex biochemical events that starts from amino acid tyrosine and its metabolite, dopa. The types and amounts of melanin produced by melanocytes are determined genetically and are influenced by a variety of extrinsic and intrinsic factors such as hormonal changes, inflammation, age and exposure to UV light. These stimuli affect the different pathways in melanogenesis. In this review we will discuss the regulatory mechanisms involved in melanogenesis and explain how intrinsic and extrinsic factors regulate melanin production. We will also explain the regulatory roles of different proteins involved in melanogenesis.

## 1. Introduction

Melanogenesis by definition is the production of the melanin pigments; these are most often produced by cells called melanocytes [1,2]. Melanocytes are dendritic cells of the neuroectoderm [1,3,4,5]. Melanoblasts, the precursor cells of melanocytes, are unpigmented cells that originate from embryonic neural crest cells [6,7]. After closure of the neural tube [8], melanoblasts migrate to various regions of the body and develop into melanocytes as well as cells of the peripheral nervous system, bone and cartilage of the head, and the choroid of the eye [1,2]. Melanoblasts that develop into melanocytes are predominantly found in the basal layer of skin epidermis and hair follicles [9,10], and can be identified by the expression of melanocyte-specific markers such as tyrosinase (TYR), tyrosinase-related protein 1 (TYRP1), DOPAchrome tautomerase or tyrosinase-related protein-2 (TYRP2), premelanosome protein 17 (Pmel17/gp1000), melan-A or melanoma antigen recognized by T cells 1 (MART-1) and microphthalmia-associated transcription factor (MITF) [11].

**Figure 1:**
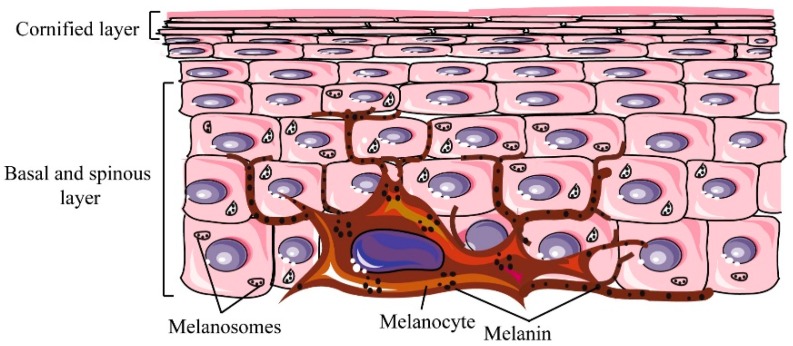
Association of keratinocytes and melanocytes. The dendritic melanocyte is located in the basal layer of skin and produces melanin. Melanin pigments in melanosomes are transferred to keratinocytes.

The primary function of melanocytes is the production of the melanin pigment. Melanocytes in the skin are surrounded by keratinocytes (one melanocyte is surrounded by approximately 36 keratinocytes) [12,13], to which they transfer their melanin pigment [13,14] (Figure 1). The molecular structure of melanin is well suited to absorbing ultraviolet (UV) [15] and visible light and thus serves as protection against UV radiation (UVR) from sunlight [16,17]. Melanocytes are also found in other tissues of the body such as the central nervous and cardiovascular system, the uvea of the eye, cochlea and even adipose tissue [18,19].

Given that melanocytes are present in a variety of tissues in the body, the initiation and extent of pigmentation or melanogenesis can be influenced by a number of intrinsic and extrinsic factors. In addition, melanogenesis can also be influenced by predetermined genetic factors such as age and ethnicity, and this process has evolved to provide protection and maintenance of homeostasis. Extrinsic factors include UVR and certain chemical compounds, while intrinsic factors include molecules secreted by surrounding keratinocytes, fibroblasts, inflammatory, neural or endocrine cells which are affected by conditions such as pregnancy and diabetes [8]. It has been proposed that the precise response of the skin to these stimulatory factors is mediated by a cutaneous neuroendocrine system (reviewed in [20]). This review will summarize the genes and molecular pathways that regulate melanogenesis and the processes that may influence this pigment-producing pathway.

## 2. Melanogenesis: Melanin Pigment Production

Melanocytes contain melanosomes, which are subcellular lysosome-like organelles in which melanin pigments are synthesized and stored [21] before distribution to the surrounding keratinocytes (Figure 1). Melanosomes require a number of specific enzymatic and structural proteins to mature and become competent in order to produce melanin. TYR and TYRP2 are among critical enzymes that affect the quantity and quality of melanin, whilst Pmel17 and MART1 are critical structural proteins [22]. AP-3, BLOC-1 and OCA2 [23] have important roles in sorting and trafficking melanosomes.

**Figure 2:**
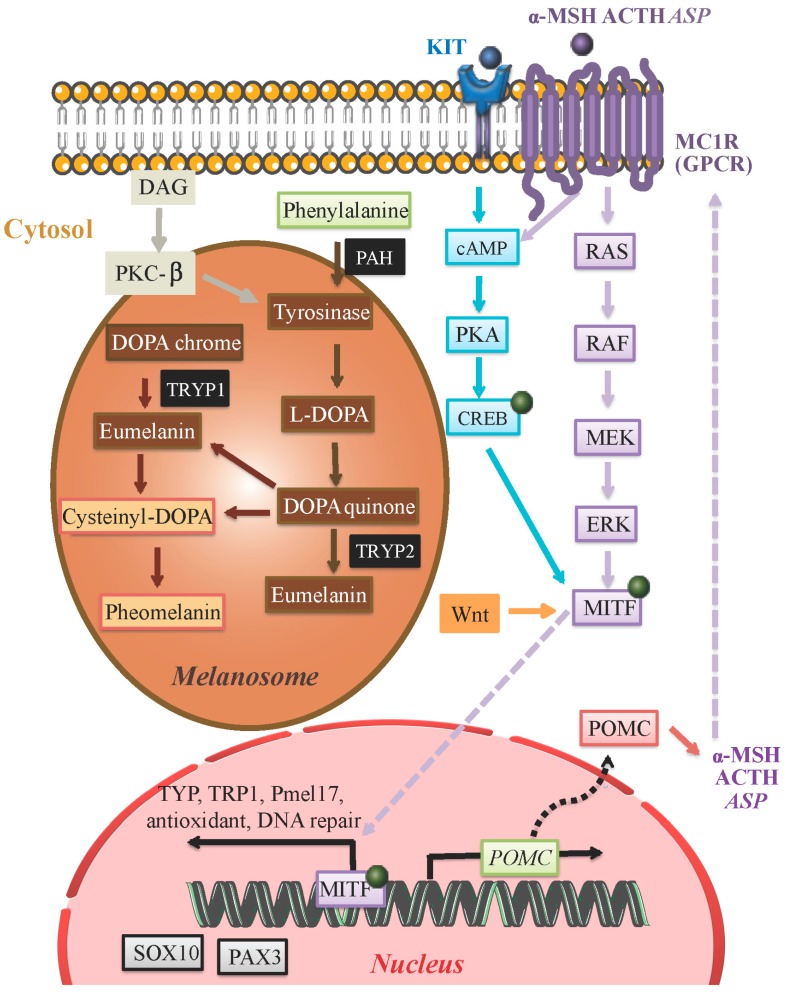
Eumelanin and pheomelanin are synthesized within melanosomes of melanocytes by a series of reactions that are catalyzed by specific melanogenic enzymes (black). Production of these enzymes is driven by the MITF transcription factor whose activity is regulated by a number of signaling pathways including PKC (brown), cAMP (blue), MEK (purple), and WNT (orange). These signaling pathways are activated upstream by receptors such as KIT (ligand: SCF) and MC1R (ligands: α-MSH, ACTH and ASP). The MITF transcription factor drives the expression of a number of genes including *SOX10* and *PAX3*. Protein kinase C (PKC); cyclic AMP (cAMP); MAPK/ERK Kinase (MEK); Wingless-related integration site (WNT); Stem Cell Factor (SCF); Melanocyte-specific melanocortin-1 receptor (MC1R); α-melanocyte-stimulating hormone (α-MSH); adrenocorticotropic hormone (ACTH); agonist stimulating protein (ASP).

Melanogenesis in prokaryotes consists of a series of reactions involving a single melanogenic enzyme [2]. In vertebrates, melanin is formed from the phenolic amino acid precursor l-tyrosine through a series of enzymatic and spontaneous chemical reactions termed the Raper–Mason pathway [2]. In mammals, a multienzyme complex constituted by melanocyte-specific gene products acts to coordinate the tightly regulated melanogenesis pathway. Mammalian melanogenic enzymes are highly similar metalloproteins: tyrosinase, TYRP1 or gp75 and TYRP2. Melanin is synthesized through a series of interactions catalyzed by these enzyme complexes (Figure 2). There are two types of melanin (synthesized from dopaquinone precursors) in mammals: brownish black eumelanin synthesized from l-dopachrome and reddish yellow pheomelanin whose synthesis is dependent on the availability of sulfhydryl compounds in melanosomes [2,24,25]. l-Dopachrome increases tyrosinase activity, whereas l-tyrosine induces melanosome synthesis, and increases tyrosinase activity [26]. l-Tyrosine serves as the starting material for biosynthesis of melanin. The immediate product, dopa, upregulates melanin synthesis [27,28]. Therefore, it is proposed that melanocytes can act as regulators for local and global homeostasis of melanogenic systems by controlling l-tyrosine levels and l-DOPA production [27,28].

A higher overall melanin density results in darker skin [16,17], but the eumelanin to pheomelanin ratio also contributes to the differences seen in human skin pigmentation [13]. Individuals with melanocytes that make more pheomelanin than eumelanin tend to have lighter skin that is more prone to blistering and burning [29,30]. Skin that has pheomelanin also produces more reactive oxygen species, which can accelerate carcinogenesis, compared with skin that produces eumelanin or has no melanin [31,32]. Following exposure to UV radiation, melanin can act as a photosensitizer to generate superoxide radicals that cause lethal cellular injury but melanin is important for skin homeostasis and tanning indicative of a distress signal [33].

Hydroxylation of l-tyrosine to l-DOPA is the rate-limiting step in melanin synthesis and is catalyzed by tyrosinase [2,8] (Figure 2), which is a copper-containing membrane-bound located in melanosomes [2,8]. l-Phenylalanine in the cytosol may be converted to tyrosine by phenylalanine hydroxylase (PAH) in order to serve as the substrate for tyrosinase [8]. Aside from tyrosinase, TYRP1, and TYRP2 are present in melanosomes and also play a crucial role in catalyzing eumelanin-producing reactions. TYRP1 has been suggested to increase the eumelanin: pheomelanin ratio and protect against oxidative stress via its peroxidase effect [8]. The biochemical pathway resulting in the formation of pheomelanin involves the synthesis of cysteinyl dopa, which is a condensation product of dopaquinone and the amino acid, l-cystein [2]. During melanogenesis tyrosine and l-DOPA serve as substrates for tyrosinase. They also act as bioregulatory agents for other cellular functions. These include dendrite formation and enhancement of cell migration (through downregulation of PKC) [28]. The pH within melanosomes can determine the rate of melanin production and eumelanin to pheomelanin ratios [8,34].

## 3. Core Molecular Pathways Influencing Melanin Production

Melanin production is initiated and regulated by a number of signaling systems and transcription factors including the tyrosine kinase receptor KIT, its ligand SCF, as well as MITF [35]. Genetic, biochemical and pharmacological evidence has established that signaling from MC1R is the main factor dictating melanogenesis [2].

The MC1R is a member of a subgroup of class A G-protein-coupled receptors that includes MC1R to MC5R [2]. Eumelanin synthesis is stimulated via the MC1R agonists α-MSH and ACTH [36] while pheomelanin synthesis is simulated via ASP [8,37] (Figure 2). α-MSH also regulates pheomelanin and eumelanin proportions via the MC1R [38] (Figure 2) [8,34]. α-MSH is cleaved from a precursor protein called pro-opiomelanocortin (POMC) produced by the pituitary gland and epidermal keratinocytes [2,33,39,40] allowing for local paracrine regulation [2]. Differential expression of *POMC* has been shown during normal physiological hair growth, immune cytokine release, the presence of cutaneous pathology or UVR exposure. UVR acts as a stimulatory factor on *POMC* gene expression, and it is suggested that UVR-triggered oxidative stress leads to POMC peptide production [41].

It is believed this signaling pathway is critically involved in physiological adaptations of the skin to environmental factors such as UV exposure. Activation of MC1R by α-MSH or ACTH increases cAMP synthesis which indirectly induces a switch from the production of pheomelanin to eumelanin synthesis [2] (Figure 2).

Along with the α-MSH-MCR1 signaling pathway, the SCF-KIT receptor tyrosine kinase pathway is involved in melanocyte pigmentation and development via the activation of the MITF transcription factor (of which the M-MITF isoform is specific to the melanocyte lineage) [1,42]. MITF-target genes regulate melanocyte pigmentation (by mechanisms that include the induction of *TYR*). Factors that influence MITF expression and activity are discussed in more detail in the following sections.

The transcriptional and post-transcriptional mechanisms governing melanogenic molecular pathways remain uncertain. Transcriptional stimulation is mainly achieved through the cAMP-dependent activation by MITF of several melanogenic genes. Upstream, MITF is phosphorylated by MAPK–ERK signaling, as well as by ribosomal S6 kinases downstream of KIT or MC1R activation. Signaling downstream of MC1R involves the activation of cAMP and of the CREB transcription factor which induces the expression of MITF. Additionally, the WNT pathway can also contribute to MITF expression [42].

A specific sequence in the *tyrosinase* gene (*TYR*) is targeted by MITF resulting in upregulation of *tyrosinase*. However, in humans, the increases in tyrosinase abundance in melanocytes are modest in response to α-MSH stimulation. It has been shown that intracellular cAMP elevating agents such as forskolin lead to upregulation of MITF protein levels without a concomitant induction of *tyrosinase* mRNA. This observation suggested that post-transcriptional regulatory mechanisms for MITF protein induction operate. Moreover, in mouse melanocytes, increases in transcription of the *TYR* gene and of the translation of its message are usually lower than the stimulation of tyrosinase specific activity. This confirms that α-MSH not only triggers the transcription of *tyrosinase* but also stimulates post-translational increases of tyrosinase activity [2].

α-MSH upregulates *TYRP2* expression, while ASIP leads to downregulation of *TYRP2*. These disparate effects suggest a role for TYRP2 as a positive regulator of eumelanogenesis. In mouse melanocytes carrying loss of function mutations in the *TYRP2* locus, the relative levels of eumelanins to pheomelanins were significantly decreased. The regulation of the pigment type produced in melanocytes might also be modulated by α-MSH due to availability of other factors such as thiol compounds [2].

Tyrosinase determines the color of mammalian skin and hair. Accumulation of this enzyme results in dermatological disorders such as melisma, age spots and actinic damage. Melanin production can be inhibited either by avoiding UV or by inhibition of melanocyte metabolism and proliferation. A number of tyrosinase inhibitors from both natural and synthetic sources have become available to date (reviewed in [43]), although further research is required to make these inhibitors available for patients suffering from undesired effects of tyrosinase.

## 4. Melanin Production in Hair Shaft

Precise interactions in the hair follicle pigmentary unit involving follicular melanocytes, keratinocytes and dermal papilla fibroblasts result in production of the hair shaft melanin components (eu- and/or pheo-melanin). Melanogenesis at the cellular level (follicular melanocyte), organ level (hair follicle), and during developmental steps are regulated by these precise interactions [44]. These steps involve the production of melanin in follicular melanocytes, the transfer of melanin granules into cortical and medullary keratinocytes, and the formation of pigmented hair shafts. In contrast to continuous melanogenesis in the skin, hair pigmentation is active only during the anagen stage (growth phase) of the hair cycle. Melanogenesis is switched off in the catagen stage (end of anagen or the transitional phase that allows the follicle to renew itself) and remains absent through telogen (dormant stage) [45]. Melanogenesis during the anagen phase is coupled with the precise regulatory network controlling hair growth, so leading to the pigmented hair shaft. The melanocyte component in hair follicles is more sensitive to aging influences than melanocytes in the epidermis [46].

There is evidence that epidermal and follicular melanins are independent units and the co-expression of white hair on highly pigmented skin can be a clear affirmation of this claim. Also melanocytes of the hair follicle produce larger melanosomes than those in the epidermis. Furthermore, follicular-melanin units are larger, more dendritic, and have more extensive Golgi and rough endoplasmic reticulum [46,47].

## 5. Regulation of Enzyme Activity in Melanogenesis

Pigmentation is known to be regulated by more than 125 distinct genes [48]. Those genes regulate key functions that are critical to melanoblasts, such as cell differentiation, and survival, as well as regulate pathways involved in pigmentation and biogenesis or function of melanosomes [22]. The following sections will discuss the molecular and genetic regulators that affect core pathways in melanogenesis.

### 5.1. α-Melanocyte-Stimulating Hormone (α-MSH)

During melanogenesis, the expression of *tyrosinase* is upregulated. The activity of tyrosinase is stimulated by α-MSH through the cAMP pathway. α-MSH binds to MC1R (melanocortin-1 receptor) on the cell surface and activates adenylate cyclase, which leads to an elevated level of intracellular cAMP (Figure 2). The expression of *tyrosinase*, *TYRP1* and *TYRP2* is induced by cAMP. Most of the biological effects of cAMP have been shown to be mediated through cAMP-dependent PKA (protein kinase A) which results in the phosphorylation of CREB [49] (Figure 2). However, it has been shown that *TYRP1* and *-2* don’t have cAMP response elements (CREs) in their promoter regions. It is believed that the regulation of these genes by cAMP occurs by the direct involvement of MITF. A 20-bp segment (positions −1861 to −1842) known as the tyrosinase distal element (TDE) within the 5′-flanking enhancer element of the human *tyrosinase* gene is responsible for pigment cell-specific transcription of *TRYP1* and *-2* [50]. MITF binds the M-box (AGTCATGTGCT) located in the TDEs of *TYRP1* and *-2* and regulates their expression. Conversely the promoter of the *MITF* gene itself contains the consensus CRE sequence, and thus its expression is likely upregulated by α-MSH induced expression of *MITF* through a cAMP-dependent pathway [51].

### 5.2. Microphthalmia-Associated Transcription Factor (MITF)

MITF is the only member of the microphthalmia family of transcription factors known to be essential for melanocyte development. The *MITF* gene contains multiple promoters, and at least nine promoter-exon units direct the expression of the gene. The M promoter that is located most proximal to the common downstream exons is selectively used in melanocytes and is targeted by several transcriptional factors including PAX3, CREB, SOX9, SOX10, lymphoid enhancer-binding factor 1 (LEF1 or TCF7L3), one cut domain 2 (ONECUT-2) and MITF itself [52,53]. *MITF* is not only regulated at the transcriptional level; it is also subject to different post-transcriptional modifications. Furthermore, mitogen-activated protein kinase (MAPK), ribosomal S6 kinase (RSK), glycogen synthase kinase-3β (GSK3β) and p38 phosphorylate MITF, modulating its transcriptional activity in response to specific environmental stimuli.

c-KIT-activated phosphorylation of MITF Ser^73^ is mediated by extracellular-signal regulated kinase 2 (ERK2) and leads to recruitment of the CREB binding protein CBP. On the other hand, MITF Ser^409^ is phosphorylated by p90 ribosomal S6 kinase (p90RSK) in melanocytes. MITF is also post transcriptionally-modified (sumoylated) by protein inhibitor of activated STAT3 (PIAS3) [52]. This modification affects MITF transcriptional activity, and the unsumoylated protein seems to be more active in contacting the promoter at multiple binding sites. These observations suggest that post-translational modifications of MITF regulate its activity.

The DNA binding and transcriptional activities of MITF are also repressed by PKC inhibitor. The interaction between MITF and PIAS3 is dictated by the phosphorylation pattern of MITF. Phosphorylation of Ser^73^ leads to increased MITF-PIAS3 interaction while Ser^409^ phosphorylation reduces this interaction [54].

**Figure 3:**
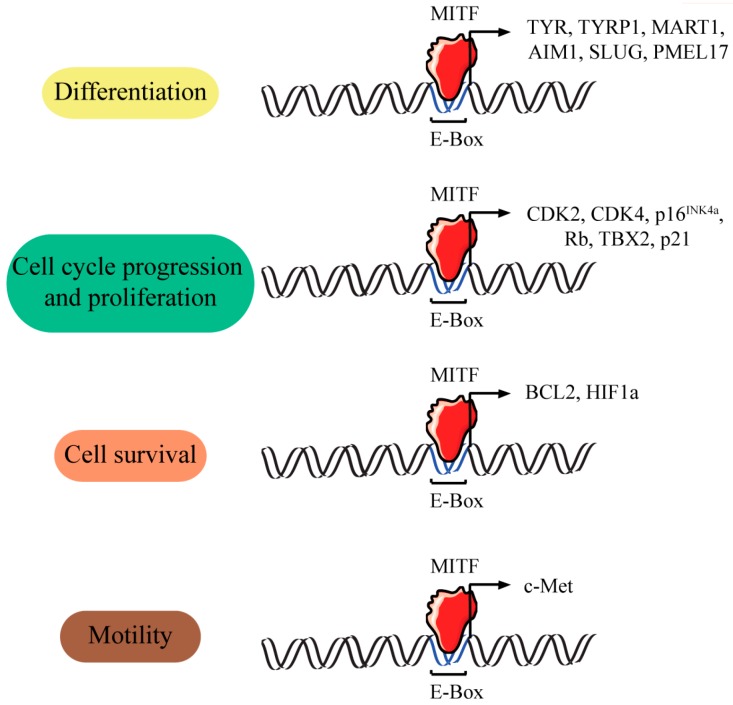
Genes activated by MITF. The transcription of multiple genes is regulated by MITF. This in turn regulates multiple cellular processes including differentiation, proliferation, survival and motility. MITF binds to specific sequences that are classifiable as members of the widely distributed E-(enhancer)-box family of regulatory motifs [52].

MITF not only regulates the transcription of three major pigmentation enzymes: *TYR*, *TYRP1* and *TYRP2* but is also involved in the regulation of several other genes with less known functions (Figure 3). The regulation of multiple pigmentation and differentiation related-genes by MITF has solidified the hypothesis that MITF functions as a central regulator of melanogenesis.

### 5.3. Wnt Regulation of MITF

Wnt signaling plays a critical role in melanocyte development. Wnt1 and Wnt3a promote the development of neural crest cells into pigment cells and without these two proteins neural crest cells cannot differentiate to melanocytes [55,56]. Wnt1 signals to melanoblasts to increase the number of melanocytes, while Wnt3a and β-catenin promote the differentiation of neural crest cells into melanocytes [57,58]. Furthermore, Wnt3a acts on melanoblasts to maintain MITF expression and promote melanoblast differentiation into melanocytes [57].

The stability of cytoplasmic β-catenin is increased by binding of Wnt proteins to their receptors and leads to transport of β-catenin into the nucleus, where it regulates transcription of *MITF* through interactions with LEF/TCF transcription factors [59]. Studies in melanocytes indicate that β-catenin and LEF1 synergistically regulate expression from the *MITF-M* promoter through the LEF1 binding sites [60] and overexpression of β-catenin induces the expression of *MITF* in melanoma [61]. LEF1, PAX3, SOX10, CREB, Onecut-2 and MITF protein itself bind to the *MITF-M* promoter and regulate *MITF* transcription [59].

### 5.4. Protein Kinase C

The protein kinase C (PKC)-dependent pathway also regulates melanogenesis [62]. PKCs are encoded by nine genes and are classified into three sub-classes based on their requirements for activation (Table 1). PKCα, PKCβ and PKCγ are activated by calcium and diacylglycerol (DAG); PKCσ, PKCθ, PKCε and PKCη are activated by DAG; while PKCζ and PKCι are not activated by DAG or calcium. The alternative splicing patterns of *PKC*s generate further heterogeneity in this family of proteins [63]. PKCß is an isoform with specific roles in melanogenesis through the phosphorylation and activation of tyrosinase [64].

Tyrosinase activity rather than total tyrosinase protein determines the level of melanin production following UV exposure. The activity of tyrosinase is dependent on the phosphorylation of serine residues 505 and 509 in its cytoplasmic domain. PKCβ is transcriptionally upregulated following UV irradiation [65] and is activated by DAG generated from UV-irradiated cell membranes, which induces its translocation from the cytoplasm to the membrane where it phosphorylates and activates tyrosinase. The association of PKC with specific subcellular fractions is dictated by structural differences among various isoforms. Receptors for activated C-kinase (RACK) control the translocation of PKC to specific cellular compartments in an isoform-specific manner. RACK-I is a specific receptor for activated PKCβ. In human melanocytes translocation of an activated PKCβ /RACK-I complex to the melanosome membrane leads to tyrosinase activation [66].

### 5.5. Sox Family

The SOX family includes about 20 transcription factors containing SRY high mobility box (HM-box) domains that mediate sequence-specific DNA binding. When SOX proteins bind to DNA, they regulate the transcription of target genes through a diversity of mechanisms (reviewed in [67]). Nine groups of SOX proteins are known in mammals (SOXA, B1, B2 and C–H). SOXE includes SOX9 and 10 which are essential developmental regulators of melanogenesis. Melanocytes originate from neuroectodermal tissue, and SOX proteins influence the differentiation of neural crest during its generation. In most species, SOX8, 9 and 10 are expressed in the dorsal neural tube and the neural crest (reviewed in [68]). Unique functions attributed to SOX10 underlie the development of the melanocyte lineage from the neural crest in mammals [69]. SOX10 controls the transcription of *MITF*, which in turn controls a set of genes critical for melanogenesis including *TYRP2*, *PMEL* and *TYRP1*. In the absence of SOX10, MITF cannot induce the expression of tyrosinase [70]. SOX10 transactivates a number of other genes needed for melanin synthesis, including *TYRP2*, which MITF cannot activate on its own [71]. The most important role of SOX9, the other member of the SOXE group, in melanoblast development may lie in its ability to induce the expression of *SOX10* [72,73]. In the dorsal neural tube, *SOX10* is upregulated around the same time as, or after *SOX9*. *SOX9* is downregulated in trunk neural crest cells in mouse, chicken, *Xenopus* and zebrafish as they become migratory [73] while *SOX10* expression persists for some time in the migratory neural crest cells. Loss of *SOX10* lead to complete absence of neural crest cells on the pigment cell migratory pathway [71,74]. Additional SOX family members, including *SOX18* and *SOX5* (of the SOXF and SOXD groups respectively) are implicated in regulating aspects of the melanocyte life cycle (reviewed in [68]).

**Figure 4:**
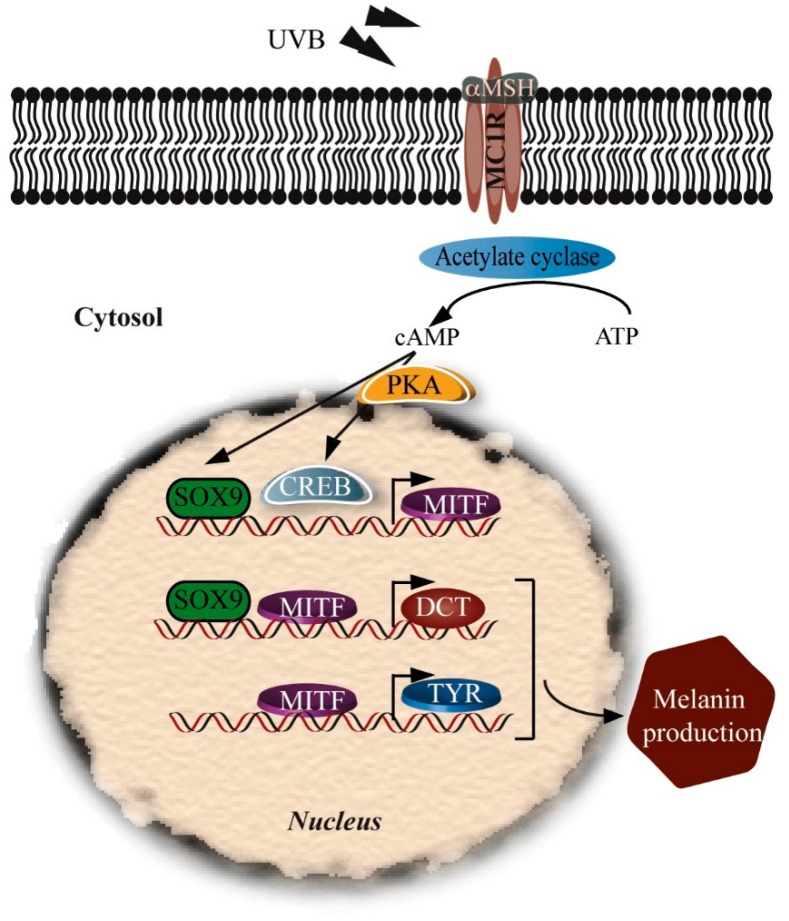
Upregulation of melanogenesis by SOX9. cAMP is elevated following UVB radiation and expression of α-MSH. α-MSH activates the PKA pathway to phosphorylate CREB and upregulate SOX9. Activated CREB and SOX9 induce *MITF* gene expression. MITF upregulation induces *TYR* expression leading to induction of melanogenesis. UVB irradiation upregulates SOX9 and activates the *MITF* promoter followed by *TYRP2* induction.

SOX proteins are not expressed only during melanoblast development. Their expression is continued after birth. *SOX10* is downregulated during melanocyte differentiation while *SOX9* is upregulated. *SOX9* may act in melanocyte differentiation in adult skin similarly to *SOX10* during embryogenesis (Figure 4). Ectopic expression of *SOX9* is sufficient to promote melanocyte differentiation [73].

Exposure to UVB upregulates *SOX9* and activates the *MITF* promoter, induces *TYRP2* and *TYR* expression, and increases melanin production [53]. *SOX9* has no UV-responsive elements in its promoter, so UV must act through intermediates to activate SOX9 expression. The activity of SOX9 in adult melanocytes is dependent on the cAMP pathway. Surrounding keratinocytes secrete α-MSH which activates melanocyte MCIRs and (via cAMP) PKA, leading to upregulation of SOX9 and CREB which regulate the *MITF* promoter. SOX9 and MITF then act together to regulate the *TYRP2* promoter, while *MITF* acts on the *TYR* promoter to upregulate melanogenesis within melanosomes (Figure 4). SOX10 cannot substitute for the SOX9 in upregulating *TYRP2* and *MITF* [53].

### 5.6. PAX3

*PAX3* acts as key player in the development of the neural crest and its derivatives, including melanocyte progenitors and is a member of the paired box family of genes. [75]. It has been shown that PAX3 is expressed in melanoma tissues, cell lines and circulating melanoma cells [76,77] as well as normal skin melanocytes and melanocytic lesions [78,79]. It is believed that PAX3 contributes to cell survival and growth in the melanocytic lineage and it is known to be important in regulating the transition from early melanoblasts derived from the neural crest to mature melanocytes [76,77]. The co-expression of PAX3 with tyrosinase and Melan-A suggested it is expressed in melanoblasts and differentiated melanocytes. PAX3 is also expressed in nevi, primary melanoma as well as metastatic melanoma at various concentrations. However its expression is not an indication of melanoma subtype, metastasis, growth phase, or Clark level and cannot be used as diagnostic marker [79]. The absence of differential expression of PAX3 in melanocytes of normal human skin, nevi, and melanomas has been also reported. These studies have suggested that in melanomas from younger individuals, PAX3 tended to be expressed more often [76,80,81]. These controversial data suggest the need for more extensive studies to investigate the role of PAX3 in normal melanogenesis and melanoma progression.

An in vitro study by Bondurand et al. [82] suggested that PAX3 acts together with SOX10 to induce the expression of *MITF*. They showed that the *MITF* promoter contains several SOX10 activation regions, [82]. The *MITF* promoter sequence also contains a PAX3 binding site which is located between positions −40 and −26. This region is known to be critical for transcription activation of MITF.

### 5.7. Melanocyte Differentiation

Mammalian melanocyte precursor cells, melanoblasts, differentiate from embryonic neural crest cells via SOX10-positive melanoblast bipotent progenitor cells. Melanoblasts are specified subsequently and express MITF, TYRP2 and KIT; they colonize the embryonic hair follicle where a proportion of melanoblasts differentiate into pigment-producing melanocytes. A subset of melanoblasts dedifferentiate (losing MITF and KIT expression but not TYRP2/DCT to form melanocyte stem cells (MelSCs) that replenish the differentiated melanocytes [83].

Maintenance of the melanocyte population is dependent on the presence of MelSCs, a quiescent population that remains present in the bulge region of the hair follicle [84,85] and that expresses TYRP2 and TYRP1, but lacks TYR expression [86]. Bcl2 expression is essential for the survival of MelSCs while MITF is essential for melanocyte stem cell maintenance by preventing premature differentiation/pigmentation [85,87]. Transforming growth factor-beta (TGF-β) also maintains MelSCs in a quiescent state [84]. Low concentrations of PAX10, high levels of TGFβ and increased activity of the Notch pathway through the Hes1 downstream transcription factor also protect MelSCs from the differentiation process [80].

Wnt ligands induce MelSCs to proliferate into melanocyte precursor cells. The Wnt signaling pathway results in MelSC differentiation into melanocytes, whereas inhibition maintains the MelSC phenotype [84]. The WNT pathway targets *PAX3*, *SOX10*, and *MITF*. PAX3 prevents terminal differentiation of MelSCs into melanocytes, a process antagonized by β-catenin [84].

### 5.8. Melanogenesis Protects against UV Damage

Traditionally the purpose of melanogenesis or skin pigmentation was believed to be for photoprotection. Melanin functions as a broadband UV absorbent, an antioxidant and a radical scavenger. There is substantial evidence for the mutagenic role of UV light and its characteristic base substitution signature (G to T for UVA, C to T for UVB) in melanoma pathogenesis [5,88,89,90]. It has been suggested that UVR is responsible for both acute and chronic photodamage of the skin and that its repair initiates signals that induce melanogenesis. Human skin responds to acute UVR exposure by photodamage, erythema, mutation, immunosuppression, synthesis of vitamin D and tanning or melanogenesis while chronic UVR exposure effects induce mutation and immunosuppression and skin response of photoaging and photocarcinogenesis [91]. Some of the most common risk factors associated with melanoma include chronic exposure to UV light [92], the occurrence of moles (nevi), age, gender (males are at higher risk), and fair skin color that tans poorly (often dictated by ethnicity) [93].

The UV activity spectrum that leads to induced melanogenesis in human skin is identical to the spectrum that induces erythema [94] and the typical DNA photoproduct cyclobutane pyrimidine dimers (CPD) [95]. Treatment of UV-exposed melanocytes with molecules that are responsible for the repair of CPD (excision enzyme T4 endonuclease V), not only increases DNA repair but also doubles melanin content [96].

Pigmentation shortens overall survival and disease-free survival in patients with metastatic melanoma [97]. Melanin also seems to protect malignant melanocytes from chemo-, radio- and photodynamic therapy [98] Melanin levels are significantly higher in invasive melanoma cells. Melanin in metastatic melanoma cells decreases the effectiveness of radiotherapy, and melanin synthesis is correlated with higher disease stage. Inhibition of melanogenesis was found to improve radiotherapy in melanoma patients [99]. In metastatic melanoma patients, it was observed that patients with melanotic tumors had significantly shorter disease-free survival and overall survival in comparison to patients with amelanotic lesions. Also, overall survival and disease-free survival is reduced in melanin-producing lymph node metastases, compared to amelanotic metastases. Therefore inhibition of melanogenesis has been suggested to be a rational approach for metastatic melanoma therapy [97].

### 5.9. Keratinocytes

Each melanocyte in the skin is surrounded by approximately 36 keratinocytes [12,13], to which it transfers its melanosomes [13,14]. The number of melanosomes in keratinocytes contributes to the differences seen in human skin pigmentation [13]. UV radiation can stimulate pigmentation by influencing melanin production (via α-MSH, discussed later), and melanocyte proliferation and distribution in the skin [4,8,19]. Pigmentation increases the resistance of the skin to subsequent sunburn [19].

Melanosomes, where melanin pigments are synthesized and stored [21], mature following an increase in extracellular pH from 5 to 6.8, before being distributed to other cells such as surrounding keratinocytes. Melanosome translocation from melanocytes to keratinocytes takes place by phagocytosis by keratinocytes [100] via the protease activated receptor-2 (PAR2) and unidentified lectins and glycoproteins, a process that is activated by UVB radiation and regulated by α-MSH [12,101]. There is evidence that p53 function may contribute to the control of melanogenesis, through a number of routes [102,103,104].

Pigment-filled melanosomes move from the perinuclear region to the tips of the melanocyte dendrites along microtubules [8,12]. From here, they translocate and disperse into keratinocyte cytoplasm eventually capping keratinocyte nuclei [12]. In darker skinned individuals, melanosomes tend to be larger, elongated and numerous, and consequently have a longer degradation time in keratinocytes. These features of melanocytes are genetically determined at birth and not influenced by extrinsic factors such as UV light [8]. UVR can however influence the number of dendrites on melanocytes and the transfer of melanosomes to keratinocytes [8].

Melanocytes and keratinocytes interact with each other extensively following extrinsic or intrinsic stimuli. In response to UVR exposure, keratinocytes produce several factors, with paracrine action on melanocytes, and these may have stimulatory or inhibitory effects on melanin production. On the other hand, melanocytes produce several factors which act in an autocrine or paracrine manner on keratinocytes, and these are known to be involved in immune and inflammatory responses [8].

Glutamate receptors have been described in both keratinocytes and melanocytes [105]. Keratinocytes are capable of producing and releasing glutamate which acts as an agonist for epidermal glutamate receptors [105]. It would be expected that melanocytes may be involved in glutamate signaling [106,107] as their natural location in the epidermis is within a bed of keratinocytes [12,13,14]. Melanocytes are sensitive to extracellular glutamate concentrations, and glutamate signaling in melanocytes is involved in their differentiation, proliferation and morphology [106]. Human melanocytes express metabotropic (GluR2, 4 and 6) and ionotropic (*N*-methyl-d-aspartate) glutamate receptors and transporters, but they neither produce nor release glutamate as a ligand [106].

### 5.10. Moles (Melanocytic Nevi)

Dysregulation of molecular pathways within melanocytes can lead to the formation of lesions such as nevi and melanoma that invade the surrounding tissue of the skin and mucosal surface [108]. Blue nevi can also form when melanocytes are rich in melanin [109]. Differences in migratory phenotype and intercellular adhesion capacities between nevus cells and normal melanocytes indicate that they may represent different melanocytic cell subpopulations. Similar levels of integrins are expressed in the melanocytes of adults and children, but there are differences in function of both α3 and α6 integrin subunits and in migratory/adhesive behaviors. This observation may suggest that there are different states of age-dependent maturation for melanocytes [110].

## 6. Melanocytes and the Inflammatory Response

Melanocytes are phagocytic cells that play a role in inflammatory responses. They respond to inflammatory events in the epidermis by producing either more or less melanin (hyperpigmentation or hypopigmentation, respectively) [19]. The pro-inflammatory cytokine interleukin-1 upregulates cutaneous levels of *POMC* mRNA, POMC peptides, and MSH receptors [39]. α-MSH downregulates the immune response following damage, preventing autoimmunity, but it also induces DNA damage repair. In addition to α-MSH, melanocytes produce other substances that regulate and enable crosstalk between different cell types in the epidermis. α-MSH and ACTH peptides produced in the epidermis induce nitric oxide production in melanocytes. Moreover, melanocortins may regulate the release of cytokines, catecholamines (CA), and serotonin (5HT) from melanocytes [40]. Melanin has also been found to counteract the effects of damage caused by reactive oxygen species in apoptotic tissue [19].

## 7. Extrinsic Factors Affecting Melanogenesis

As described above, the main extrinsic regulatory pathway in melanogenesis involves the MC1R receptor, which is activated by αMSH and acts through cAMP. However, there are other melanocyte receptors such as muscarinic receptors and α and β estrogen receptors, which are associated with adenyl cyclase and cAMP production. The increase in estrogen levels during pregnancy also can cause hyperpigmentation [111]. Melanogenesis via the cAMP and PKC-β pathways can be stimulated by catecholamines produced by keratinocytes from L-DOPA, which bind to α1 and β2 adrenergic receptors in melanocytes. Norepinephrine (acting through α1 adrenergic receptors) and ACTH 1-17 (acting though MC1Rs) can activate the inositol trisphosphate/diacylglycerol (IP3/DAG) pathway, which promotes the release of calcium in the cytoplasm of melanocytes. DAG is an activator of PKC-β which phosphorylates tyrosinase and induces melanogenesis; DAG can also be released from melanocytes through UVR action in the lipid membrane (reviewed in [8]).

It is reported that melatonin acts to protect cells against UVR-induced damage, at least partly by DNA repair through receptor-independent mechanisms, or by activation of putative melatonin nuclear receptors. It has been proposed that endogenous intracutaneous melatonin combined with topically-applied exogenous melatonin may act as a potent anti-oxidative defense system against UV-induced damage to the skin (reviewed in [112]).

Other extrinsic factors that can influence melanogenesis include skin whitening products that may be used for clinical treatment of pigmentary disorders such as melasma or post-inflammatory hyperpigmentation. These agents are competitive inhibitors of tyrosinase itself, or of tyrosinase maturation, and are also inhibitors of melanin transfer via melanosomes from melanocytes to keratinocytes. Targeting upstream (of tyrosinase) components of melanogenic pathways such as SOX9, MITF and PKC show similar effects on tanning in animal models, indicating that extrinsic factors also interfere with these core signaling pathways [113]. Magnesium-l-ascorbyl-2-phosphate (MAP), hydroxyanisole, *N*-acetyl-4-*S*-cysteaminylphenol, arbutin (hydroquinone-β-d-glucopyranoside) and hydroquinone (HQ) are the most widely prescribed skin-lightening and depigmenting agents (reviewed in [114]). Amikacin is an aminoglycoside antibiotic that inhibits melanin biosynthesis in a concentration-dependent manner and induces concentration-dependent loss of melanocytes viability. Its mechanism involves decreasing cellular tyrosinase activity [115]. On the other hand thioridazine, an antipsychotic agent that induces ocular and skin disorders. It also has anticancer and antibacterial effects, and increases apoptosis in melanoma cells [116]. Thioridazine induces oxidative stress by affecting the cellular antioxidant defense system and, at higher concentrations, it inhibits melanogenesis. This suggests an important role of reactive oxygen species in mechanisms of the side effects induced by this drug in pigmented cells [117].

## 8. Conclusions

Factors driving melanocyte function, whether intrinsic or extrinsic, are genetically regulated but are further influenced by differences in the post-transcriptional regulation of melanogenesis-related genes. These regulatory mechanisms not only influence melanocyte development and differentiation but also melanin production. Core pathways that dictate melanocyte development and melanin production, as discussed here, are often dysregulated in skin and other melanocyte-related pathologies. Here we have described how melanogenesis in humans requires a diverse range of pathways and enzymes. These in turn require extensive regulatory mechanisms to control their function. Many factors regulate melanoblast differentiation and melanin production and a tight balance between them is essential for the function of melanocytes. Despite extensive studies in this area, many aspects of melanoblast differentiation, and melanin production in normal and disease conditions have yet to be clarified. Further study of these transcriptional mechanisms is warranted in order to better understand factors influencing melanogenesis.

Recent advances in the technologies of genome sequencing, transcriptome analysis and epigenetic analysis in normal and diseased skin have promoted our understanding of melanogenesis. These data have provided information that allows further dissection of the cell signaling pathways involved in the control of melanogenesis, and have provided much new information that should lead to improvements in our diagnostic and therapeutic capacities to treat melanin pigmentary disorders. Given the fact that melanocytes and melanins have central roles in the natural photoprotection of the skin, inflammatory responses and many intrinsic and extrinsic factors could influence their function. Therefore better understanding of the mechanisms controlling melanogenesis is required. New and safe strategies should be provided to correct abnormal melanogenesis and to improve the skin's response to the deleterious effects of chronic and excessive exposure to sunlight and other stimuli. Here we have described the major pathways that stimulate melanogenesis in the hope that a better understanding of these complex intertwined pathways will inform future therapeutics of skin disorders of melanin pigmentation.

## Figures and Tables

**Table 1:** The different subclasses of PKC. The associated genes and cofactors and their presence in (+) and absence from (−) melanocytes are listed.

Subclasses	Gene	Protein	Cofactors	Melanocytes
Classical	*PRKCA*	PKCα	Ca^2+^, DAG	+
	*PRKCB*	PKCβ		+
	*PRKCG*	PKCγ		−
Novel	*PRKCD*	PKCσ	DAG	+
	*PRKCQ*	PKCθ		−
	*PRKCE*	PKCε		+
	*PRKCH*	PKCη		+
Atypical	*PRKCI*	PKCζ	Lipids	+
	*PRKCZ*	PKCι		+

## References

[1] Bonaventure J., Domingues M.J., Larue L. (2013). Cellular and molecular mechanisms controlling the migration of melanocytes and melanoma cells. Pigment Cell Melanoma Res..

[2] Borovanský J., Wiley I. (2011). Melanins and Melanosomes Biosynthesis, Biogenesis, Physiological, and Pathological Functions.

[3] NCI N.C.I. Melanoma. http://www.cancer.gov/cancertopics/types/melanoma.

[4] Tadokoro T., Yamaguchi Y., Batzer J., Coelho S.G., Zmudzka B.Z., Miller S.A., Wolber R., Beer J.Z., Hearing V.J. (2005). Mechanisms of skin tanning in different racial/ethnic groups in response to ultraviolet radiation. J. Investig. Dermatol..

[5] Schadendorf D., Fisher D.E., Garbe C., Gershenwald J.E., Grob J.-J., Halpern A., Herlyn M., Marchetti M.A., McArthur G., Ribas A. (2015). Melanoma. Nat. Rev. Dis. Primers.

[6] Lei T.C., Virador V., Yasumoto K.-I., Vieira W.D., Toyofuku K., Hearing V.J. (2002). Stimulation of melanoblast pigmentation by 8-methoxypsoralen: The Involvement of microphthalmia-associated transcription factor, the protein kinase a signal pathway, and proteasome-mediated degradation. J. Investig. Dermatol..

[7] Sviderskaya E.V., Hill S.P., Balachandar D., Barsh G.S., Bennett D.C. (2001). Agouti signaling protein and other factors modulating differentiation and proliferation of immortal melanoblasts. Dev. Dyn..

[8] Videira I.F., Moura D.F., Magina S. (2013). Mechanisms regulating melanogenesis. An. Bras. Dermatol..

[9] Gibbs S., Murli S., de Boer G., Mulder A.A.T., Mommaas A.M., Ponec M. (2000). Melanosome Capping of keratinocytes in pigmented reconstructed epidermis—effect of ultraviolet radiation and 3-isobutyl-1-methyl-xanthine on melanogenesis. Pigment Cell Res..

[10] Cichorek M., Wachulska M., Stasiewicz A., Tymińska A. (2013). Skin melanocytes: Biology and development. Postȩpy Dermatol. I Alergol..

[11] Passeron T., Coelho S.G., Miyamura Y., Takahashi K., Hearing V.J. (2007). Immunohistochemistry and in situ hybridization in the study of human skin melanocytes. Exp. Dermatol..

[12] Seiberg M. (2001). Keratinocyte-melanocyte interactions during melanosome transfer. Pigment Cell Res..

[13] Lin J.Y., Fisher D.E. (2007). Melanocyte biology and skin pigmentation. Nature.

[14] Delevoye C. (2014). Melanin transfer: The keratinocytes are more than gluttons. J. Investig. Dermatol..

[15] Amico-Ruvio S.A., Paganelli M.A., Myers J.M., Popescu G.K. (2012). Ifenprodil effects on GluN2B-containing glutamate receptors. Mol. Pharmacol..

[16] Anderson R.R., Parrish J.A. (1981). The optics of human skin. J. Investig. Dermatol..

[17] Haider S., Cho D., Amelard R., Wong A., Clausi D.A. Enhanced classification of malignant melanoma lesions via the integration of physiological features from dermatological photographs. Proceedings of the 2014 36th Annual International Conference of the IEEE Engineering in Medicine and Biology Society (EMBC).

[18] Bastian B.C. (2014). The molecular pathology of melanoma: An integrated taxonomy of melanocytic neoplasia. Annu. Rev. Pathol..

[19] Plonka P.M., Passeron T., Brenner M., Tobin D.J., Shibahara S., Thomas A., Slominski A., Kadekaro A.L., Hershkovitz D., Peters E. (2009). What are melanocytes really doing all day long …?. Exp. Dermatol..

[20] Slominski A., Zmijewski M., Skobowiat C., Zbytek B., Slominski R., Steketee J. (2012). Sensing the environment: Regulation of local and global homeostasis by the skin neuroendocrine system. Adv. Anat. Embryol. Cell Biol..

[21] Marks M.S., Seabra M.C. (2001). The melanosome: Membrane dynamics in black and white. Nat. Rev. Mol. Cell Biol..

[22] Yamaguchi Y., Brenner M., Hearing V.J. (2007). The regulation of skin pigmentation. J. Biol. Chem..

[23] Sitaram A., Marks M.S. (2012). Mechanisms of protein delivery to melanosomes in pigment cells. Physiology.

[24] Thody A.J., Higgins E.M., Wakamatsu K., Ito S., Burchill S.A., Marks J.M. (1991). Pheomelanin as well as eumelanin is present in human epidermis. J. Investig. Dermatol..

[25] Lamoreux M.L., Wakamatsu K., Ito S. (2001). Interaction of major coat color gene functions in mice as studied by chemical analysis of eumelanin and pheomelanin. Pigment Cell Res..

[26] Slominski A., Moellmann G., Kuklinska E., Bomirski A., Pawelek J. (1988). Positive regulation of melanin pigmentation by two key substrates of the melanogenic pathway, l-tyrosine and l-DOPA. J. Cell Sci..

[27] Slominski A., Moellmann G., Kuklinska E. (1989). l-Tyrosine, l-DOPA, and tyrosinase as positive regulators of the subcellular apparatus of melanogenesis in bomirski Ab amelanotic melanoma cells. Pigment Cell Res..

[28] Slominski A., Zmijewski M., Pawelek J. (2012). l-Tyrosine and l-dihydroxyphenylalanine as hormone-like regulators of melanocyte functions. Pigment Cell Melanoma Res..

[29] Gupta S. (2014). Skin colour: No hiding in the dark. Nature.

[30] Wu S., Han J., Laden F., Qureshi A.A. (2014). Long-term ultraviolet flux, other potential risk factors, and skin cancer risk: A cohort study. Cancer Epidemiol. Biomark. Prev..

[31] Okazaki S., Funasaka Y., Wakamatsu K., Kawana S., Saeki H. (2015). Effect of infrared radiation A on photoaged hairless mice harboring eumelanin and pheomelanin in the epidermis. J. Dermatol..

[32] Chedekel M.R., Agin P.P., Sayre R.M. (1980). Photochemistry of pheomelanin: Action spectrum for superoxide production. Photochem. Photobiol..

[33] Slominski A., Tobin D., Shibahara S., Wortsman J. (2004). Melanin pigmentation in mammalian skin and its hormonal regulation. Physiol. Rev..

[34] Ancans J., Tobin D.J., Hoogduijn M.J., Smit N.P., Wakamatsu K., Thody A.J. (2001). Melanosomal pH controls rate of melanogenesis, eumelanin/phaeomelanin ratio and melanosome maturation in melanocytes and melanoma cells. Exp. Cell Res..

[35] Hou L., Panthier J.J., Arnheiter H. (2000). Signaling and transcriptional regulation in the neural crest-derived melanocyte lineage: Interactions between KIT and MITF. Development.

[36] Millington G.W. (2006). Proopiomelanocortin (POMC): The cutaneous roles of its melanocortin products and receptors. Clin. Exp. Dermatol..

[37] Le Pape E., Passeron T., Giubellino A., Valencia J.C., Wolber R., Hearing V.J. (2009). Microarray analysis sheds light on the dedifferentiating role of agouti signal protein in murine melanocytes via the Mc1r. Proc. Natl. Acad. Sci. USA.

[38] Valverde P., Healy E., Jackson I., Rees J.L., Thody A.J. (1995). Variants of the melanocyte-stimulating hormone receptor gene are associated with red hair and fair skin in humans. Nat. Genet..

[39] Slominski A., Wortsman J., Luger T., Paus R., Solomon S. (2000). Corticotropin releasing hormone and proopiomelanocortin involvement in the cutaneous response to stress. Physiol. Rev..

[40] Tsatmali M., Ancans J., Thody A.J. (2002). Melanocyte function and its control by melanocortin peptides. J. Histochem. Cytochem..

[41] Slominski A., Zmijewski M., Zbytek B., Tobin D., Theoharides T., Rivier J. (2013). Key role of CRF in the skin stress response system. Endocr. Rev..

[42] Flaherty K.T., Hodi F.S., Fisher D.E. (2012). From genes to drugs: Targeted strategies for melanoma. Nat. Rev. Cancer.

[43] Kim Y.J., Uyama H. (2005). Tyrosinase inhibitors from natural and synthetic sources: Structure, inhibition mechanism and perspective for the future. Cell. Mol. Life Sci..

[44] Slominski A., Wortsman J., Plonka P.M., Schallreuter K.U., Paus R., Tobin D.J. (2005). Hair follicle pigmentation. J. Investig. Dermatol..

[45] Slominski A., Paus R. (1993). Melanogenesis is coupled to murine anagen: Toward new concepts for the role of melanocytes and the regulation of melanogenesis in hair growth. J. Investig. Dermatol..

[46] Tobin D.J., Paus R. (2001). Graying: Gerontobiology of the hair follicle pigmentary unit. Exp. Gerontol..

[47] Tobin D.J., Bystryn J.C. (1996). Different populations of melanocytes are present in hair follicles and epidermis. Pigment Cell Res..

[48] Bennett D.C., Lamoreux M.L. (2003). The color loci of mice—A genetic century. Pigment Cell Res..

[49] Edelman A.M., Blumenthal D.K., Krebs E.G. (1987). Protein serine threonine kinases. Annu. Rev. Biochem..

[50] Yasumoto K., Yokoyama K., Shibata K., Tomita Y., Shibahara S. (1994). Microphthalmia-associated transcription factor as a regulator for melanocyte-specific transcription of the human tyrosinase gene. Mol. Cell. Biol..

[51] Bertolotto C., Abbe P., Hemesath T.J., Bille K., Fisher D.E., Ortonne J.P., Ballotti R. (1998). Microphthalmia gene product as a signal transducer in cAMP-induced differentiation of melanocytes. J. Cell. Biol..

[52] Levy C., Khaled M., Fisher D.E. (2006). MITF: Master regulator of melanocyte development and melanoma oncogene. Trends Mol. Med..

[53] Passeron T., Valencia J.C., Bertolotto C., Hoashi T., Le Pape E., Takahashi K., Ballotti R., Hearing V.J. (2007). SOX9 is a key player in ultraviolet B-induced melanocyte differentiation and pigmentation. Proc. Natl. Acad. Sci. USA.

[54] Levy C., Nechushtan H., Razin E. (2002). A new role for the STAT3 inhibitor, PIAS3: A repressor of microphthalmia transcription factor. J. Biol. Chem..

[55] Dorsky R.I., Moon R.T., Raible D.W. (1998). Control of neural crest cell fate by the Wnt signalling pathway. Nature.

[56] Dorsky R.I., Raible D.W., Moon R.T. (2000). Direct regulation of nacre, a zebrafish MITF homolog required for pigment cell formation, by the Wnt pathway. Genes Dev..

[57] Dunn K.J., Brady M., Ochsenbauer-Jambor C., Snyder S., Incao A., Pavan W.J. (2005). WNT1 and WNT3a promote expansion of melanocytes through distinct modes of action. Pigment Cell Res..

[58] Jin E.J., Erickson C.A., Takada S., Burrus L.W. (2001). Wnt and BMP signaling govern lineage segregation of melanocytes in the avian embryo. Dev. Biol..

[59] Steingrimsson E., Copeland N.G., Jenkins N.A. (2004). Melanocytes and the microphthalmia transcription factor network. Annu. Rev. Genet..

[60] Takeda K., Yasumoto K., Takada R., Takada S., Watanabe K., Udono T., Saito H., Takahashi K., Shibahara S. (2000). Induction of melanocyte-specific microphthalmia-associated transcription factor by Wnt-3a. J. Biol. Chem..

[61] Widlund H.R., Horstmann M.A., Price E.R., Cui J., Lessnick S.L., Wu M., He X., Fisher D.E. (2002). Beta-catenin-induced melanoma growth requires the downstream target Microphthalmia-associated transcription factor. J. Cell Biol..

[62] Gordon P.R., Gilchrest B.A. (1989). Human melanogenesis is stimulated by diacylglycerol. J. Investig. Dermatol..

[63] Denning M.F. (2012). Specifying protein kinase C functions in melanoma. Pigment Cell Melanoma Res..

[64] Park H.Y., Perez J.M., Laursen R., Hara M., Gilchrest B.A. (1999). Protein kinase C-β activates tyrosinase by phosphorylating serine residues in its cytoplasmic domain. J. Biol. Chem..

[65] Park H.Y., Wu C., Yonemoto L., Murphy-Smith M., Wu H., Stachur C.M., Gilchrest B.A. (2006). MITF mediates cAMP-induced protein kinase C-beta expression in human melanocytes. Biochem. J..

[66] Bae-Harboe Y.S., Park H.Y. (2012). Tyrosinase: A central regulatory protein for cutaneous pigmentation. J. Investig. Dermatol..

[67] Wegner M. (2010). All purpose Sox: The many roles of Sox proteins in gene expression. Int. J. Biochem. Cell Biol..

[68] Harris M.L., Baxter L.L., Loftus S.K., Pavan W.J. (2010). Sox proteins in melanocyte development and melanoma. Pigment Cell Melanoma Res..

[69] Kellerer S., Schreiner S., Stolt C.C., Scholz S., Bosl M.R., Wegner M. (2006). Replacement of the Sox10 transcription factor by Sox8 reveals incomplete functional equivalence. Development.

[70] Hou L., Arnheiter H., Pavan W.J. (2006). Interspecies difference in the regulation of melanocyte development by SOX10 and MITF. Proc. Natl. Acad. Sci. USA.

[71] Potterf S.B., Mollaaghababa R., Hou L., Southard-Smith E.M., Hornyak T.J., Arnheiter H., Pavan W.J. (2001). Analysis of SOX10 function in neural crest-derived melanocyte development: SOX10-dependent transcriptional control of dopachrome tautomerase. Dev. Biol..

[72] Aoki Y., Saint-Germain N., Gyda M., Magner-Fink E., Lee Y.H., Credidio C., Saint-Jeannet J.P. (2003). Sox10 regulates the development of neural crest-derived melanocytes in Xenopus. Dev. Biol..

[73] Cheung M., Briscoe J. (2003). Neural crest development is regulated by the transcription factor Sox9. Development.

[74] Dutton K.A., Pauliny A., Lopes S.S., Elworthy S., Carney T.J., Rauch J., Geisler R., Haffter P., Kelsh R.N. (2001). Zebrafish colourless encodes sox10 and specifies non-ectomesenchymal neural crest fates. Development.

[75] Tassabehji M., Read A.P., Newton V.E., Harris R., Balling R., Gruss P., Strachan T. (1992). Waardenburg’s syndrome patients have mutations in the human homologue of the *Pax-3* paired box gene. Nature.

[76] Scholl F.A., Kamarashev J., Murmann O.V., Geertsen R., Dummer R., Schafer B.W. (2001). PAX3 is expressed in human melanomas and contributes to tumor cell survival. Cancer Res..

[77] He S.J., Stevens G., Braithwaite A.W., Eccles M.R. (2005). Transfection of melanoma cells with antisense PAX3 oligonucleotides additively complements cisplatin-induced cytotoxicity. Mol. Cancer Ther..

[78] Gershon T.R., Oppenheimer O., Chin S.S., Gerald W.L. (2005). Temporally regulated neural crest transcription factors distinguish neuroectodermal tumors of varying malignancy and differentiation. Neoplasia.

[79] He S., Yoon H.S., Suh B.J., Eccles M.R. (2010). PAX3 is extensively expressed in benign and malignant tissues of the melanocytic lineage in humans. J. Investig. Dermatol..

[80] Kubic J.D., Young K.P., Plummer R.S., Ludvik A.E., Lang D. (2008). Pigmentation PAX-ways: The role of Pax3 in melanogenesis, melanocyte stem cell maintenance, and disease. Pigment Cell Melanoma Res..

[81] Plummer R.S., Shea C.R., Nelson M., Powell S.K., Freeman D.M., Dan C.P., Lang D. (2008). PAX3 expression in primary melanomas and nevi. Mod. Pathol..

[82] Bondurand N., Pingault V., Goerich D.E., Lemort N., Sock E., Le Caignec C., Wegner M., Goossens M. (2000). Interaction among *SOX10*, *PAX3* and *MITF*, three genes altered in Waardenburg syndrome. Hum. Mol. Genet..

[83] Mort R.L., Jackson I.J., Patton E.E. (2015). The melanocyte lineage in development and disease. Development.

[84] Lee J.H., Fisher D.E. (2014). Melanocyte stem cells as potential therapeutics in skin disorders. Expert Opin. Biol. Ther..

[85] Nishimura E.K., Jordan S.A., Oshima H., Yoshida H., Osawa M., Moriyama M., Jackson I.J., Barrandon Y., Miyachi Y., Nishikawa S.-I. (2002). Dominant role of the niche in melanocyte stem-cell fate determination. Nature.

[86] Gola M., Czajkowski R., Bajek A., Dura A., Drewa T. (2012). Melanocyte stem cells: Biology and current aspects. Med. Sci. Monit..

[87] Nishimura E.K., Suzuki M., Igras V., Du J., Lonning S., Miyachi Y., Roes J., Beermann F., Fisher D.E. (2010). Key roles for transforming growth factor β in melanocyte stem cell maintenance. Cell Stem Cell.

[88] Hodis E., Watson I.R., Kryukov G.V., Arold S.T., Imielinski M., Theurillat J.P., Nickerson E., Auclair D., Li L., Place C. (2012). A landscape of driver mutations in melanoma. Cell.

[89] Alexandrov L.B., Nik-Zainal S., Wedge D.C., Aparicio S.A., Behjati S., Biankin A.V., Bignell G.R., Bolli N., Borg A., Borresen-Dale A.L. (2013). Signatures of mutational processes in human cancer. Nature.

[90] Berger M.F., Hodis E., Heffernan T.P., Deribe Y.L., Lawrence M.S., Protopopov A., Ivanova E., Watson I.R., Nickerson E., Ghosh P. (2012). Melanoma genome sequencing reveals frequent PREX2 mutations. Nature.

[91] Brenner M., Hearing V.J. (2008). The protective role of melanin against UV damage in human skin. Photochem. Photobiol..

[92] Leiter U., Garbe C. (2008). Epidemiology of melanoma and nonmelanoma skin cancer—The role of sunlight. Adv. Exp. Med. Biol..

[93] Gloster H.M., Neal K. (2006). Skin cancer in skin of color. J. Am. Acad. Dermatol..

[94] Parrish J.A., Jaenicke K.F., Anderson R.R. (1982). Erythema and melanogenesis action spectra of normal human skin. Photochem. Photobiol..

[95] Young A.R., Chadwick C.A., Harrison G.I., Nikaido O., Ramsden J., Potten C.S. (1998). The similarity of action spectra for thymine dimers in human epidermis and erythema suggests that DNA is the chromophore for erythema. J. Investig. Dermatol..

[96] Gilchrest B.A., Zhai S., Eller M.S., Yarosh D.B., Yaar M. (1993). Treatment of human melanocytes and S91 melanoma cells with the DNA repair enzyme T4 endonuclease V enhances melanogenesis after ultraviolet irradiation. J. Investig. Dermatol..

[97] Brożyna A.A., Jóźwicki W., Carlson J.A., Slominski A.T. (2013). Melanogenesis affects overall and disease-free survival in patients with stage III and IV melanoma. Hum. Pathol..

[98] Slominski A., Paus R., Mihm M.C. (1998). Inhibition of melanogenesis as an adjuvant strategy in the treatment of melanotic melanomas: Selective review and hypothesis. Anticancer Res..

[99] Brożyna A.A., Jóźwicki W., Roszkowski K., Filipiak J., Slominski A.T. (2016). Melanin content in melanoma metastases affects the outcome of radiotherapy. Oncotarget.

[100] Wu X., Hammer J.A. (2014). Melanosome transfer: It is best to give and receive. Curr. Opin. Cell Biol..

[101] Virador V.M., Muller J., Wu X., Abdel-Malek Z.A., Yu Z.-X., Ferrans V.J., Kobayashi N., Wakamatsu K., Ito S., Hammer J.A. (2002). Influence of α-melanocyte-stimulating hormone and ultraviolet radiation on the transfer of melanosomes to keratinocytes. FASEB J..

[102] Hyter S., Coleman D.J., Ganguli-Indra G., Merrill G.F., Ma S., Yanagisawa M., Indra A.K. (2013). Endothelin-1 is a transcriptional target of p53 in epidermal keratinocytes and regulates ultraviolet-induced melanocyte homeostasis. Pigment Cell Melanoma Res..

[103] Unal B., Alan S., Bassorgun C.I., Karakas A.A., Elpek G.O., Ciftcioglu M.A. (2015). The divergent roles of growth differentiation factor-15 (GDF-15) in benign and malignant skin pathologies. Arch. Dermatol. Res..

[104] Lim H.-S., Jin S., Yun S.J. (2016). Modulation of Melanogenesis by heme oxygenase-1 via p53 in normal human melanocytes. Chonnam Med. J..

[105] Fischer M., Glanz D., Urbatzka M., Brzoska T., Abels C. (2009). Keratinocytes: A source of the transmitter l-glutamate in the epidermis. Exp. Dermatol..

[106] Hoogduijn M.J., Hitchcock I.S., Smit N.P., Gillbro J.M., Schallreuter K.U., Genever P.G. (2006). Glutamate receptors on human melanocytes regulate the expression of MiTF. Pigment Cell Res..

[107] Ogundele O.M., Okunnuga A.A., Fabiyi T.D., Olajide O.J., Akinrinade I.D., Adeniyi P.A., Ojo A.A. (2014). NMDA-R inhibition affects cellular process formation in Tilapia melanocytes; a model for pigmented adrenergic neurons in process formation and retraction. Metab. Brain Dis..

[108] Balois T., Chatelain C., Ben Amar M. (2014). Patterns in melanocytic lesions: Impact of the geometry on growth and transport inside the epidermis. J. R. Soc. Interface.

[109] Makker J., Sakam S., Arety P., Niazi M., Balar B. (2015). Rectal blue nevus: Case report of a rare entity and literature review. Pathol. Res. Pract..

[110] Mengeaud V., Grob J.J., Bongrand P., Richard M.A., Hesse S., Bonerandi J.J., Verrando P. (1996). Adhesive and migratory behaviors of nevus cells differ from those of epidermal melanocytes and are not linked to the histological type of nevus. J. Investig. Dermatol..

[111] Wong R.C., Ellis C.N. (1984). Physiologic skin changes in pregnancy. J. Am. Acad. Dermatol..

[112] Slominski A.T., Kleszczynski K., Semak I., Janjetovic Z., Zmijewski M.A., Kim T.K., Slominski R.M., Reiter R.J., Fischer T.W. (2014). Local melatoninergic system as the protector of skin integrity. Int. J. Mol. Sci..

[113] Smit N., Vicanova J., Pavel S. (2009). The hunt for natural skin whitening agents. Int. J. Mol. Sci..

[114] Parvez S., Kang M., Chung H.S., Cho C., Hong M.C., Shin M.K., Bae H. (2006). Survey and mechanism of skin depigmenting and lightening agents. Phytother. Res..

[115] Wrzesniok D., Beberok A., Otreba M., Buszman E. (2013). Modulation of melanogenesis and antioxidant defense system in melanocytes by amikacin. Toxicol. In Vitro.

[116] Gil-Ad I., Shtaif B., Levkovitz Y., Dayag M., Zeldich E., Weizman A. (2004). Characterization of phenothiazine-induced apoptosis in neuroblastoma and glioma cell lines: Clinical relevance and possible application for brain-derived tumors. J. Mol. Neurosci..

[117] Otreba M., Beberok A., Wrzesniok D., Rok J., Buszman E. (2015). Effect of thioridazine on antioxidant status of HEMn-DP melanocytes. Naunyn-Schmiedeb. Arch. Pharmacol..

